# Association of Facial Paralysis With Perceptions of Personality and Physical Traits

**DOI:** 10.1001/jamanetworkopen.2020.5495

**Published:** 2020-06-24

**Authors:** Keon M. Parsa, Melyssa Hancock, Peter L. Nguy, Hayden M. Donalek, Haijun Wang, Jodi Barth, Michael J. Reilly

**Affiliations:** 1 Medstar Georgetown University Hospital, Washington, DC; 2 Virginia Commonwealth University Medical Center, Richmond; 3 Georgetown University School of Medicine, Washington, DC; 4 Department of Biostatistics and Biomedical Informatics, MedStar Health Research Institute, Hyattsville, Maryland; 5 The Center for Facial Recovery, Rockville, Maryland

## Abstract

**Importance:**

Facial paralysis has a significant effect on affect display, with the most notable deficit being patients’ the inability to smile in the same way as those without paralysis. These impairments may result in undesirable judgements of personal qualities, thus leading to a significant social penalty in those who have the condition.

**Objective:**

To quantify the association of facial paralysis with the way smiling patients are perceived by others with respect to personality traits, attractiveness, and femininity or masculinity and to evaluate the potential association of facial palsy–related patient-reported outcome measures with how patients are perceived by others.

**Design, Setting, and Participants:**

This retrospective cross-sectional study used 20 images of smiling patients with facial paralysis evaluated between January 1, 2014, and December 31, 2016. Using photograph editing software, the photographs were edited to create a simulated nonparalysis smiling facial appearance. A total of 40 photographs were split into 4 groups of 10 photographs, each with 5 altered and 5 unaltered photographs. The surveys were designed such that altered and unaltered photographs of the same patient were not placed in the same survey to avoid recall bias. Anonymous raters used a 7-point Likert scale to rate their perception of each patient’s personality traits (ie, aggressiveness, likeability, and trustworthiness), attractiveness, and femininity or masculinity based on photographs in their assigned survey. Raters were blinded to study intent. Scores from the Facial Clinimetric Evaluation questionnaire were included to assess self-perception. Data were analyzed from November 11, 2019, to February 20, 2020.

**Main Outcomes and Measures:**

Ratings of personality traits, attractiveness, and femininity or masculinity. Social function domain scores and overall scores were analyzed from the Facial Clinimetric Evaluation questionnaire.

**Results:**

This study included photographs of 20 patients with facial paralysis (mean [range] age, 54 [28-69] years; 15 [75%] women). A total of 122 respondents completed the survey (71 [61%] women). Most respondents were between the ages of 25 and 34 years (79 participants [65%]). Overall, smiling photos of patients with facial paralysis were perceived as significantly less likeable (difference, −0.29; 95% CI, −0.43 to −0.14), trustworthy (difference, −0.25; 95% CI, −0.39 to −0.11), attractive (difference, −0.47; 95% CI, −0.62 to −0.32), and feminine or masculine (difference, −0.21; 95% CI, −0.38 to −0.03) compared with their simulated preparalysis photographs. When analyzed by sex, smiling women with facial paralysis experienced lower ratings for likeability (difference, −0.34; 95% CI, −0.53 to −0.16), trustworthiness (difference, −0.24; 95% CI, −0.43 to −0.06), attractiveness (difference, −0.74; 95% CI, −0.94 to −0.55), and femininity (difference, −0.35; 95% CI, −0.58 to −0.13). However, smiling men with facial paralysis only received significantly lower ratings for likeability (difference, −0.24; 95% CI, −0.47 to −0.01) and trustworthiness (difference, −0.30; 95% CI, −0.53 to −0.07). As patients’ self-reported social function and total Facial Clinimetric Evaluation scores increased, there was an increase in perceived trustworthiness (*r*s[480] = 0.11; *P* = .02) and attractiveness (*r*s[478] = 0.10; *P* = .04) scores by raters.

**Conclusions and Relevance:**

In this study, photographs of patients with facial paralysis received lower ratings for several personality and physical traits compared with digitally edited images with no facial paralysis. These findings suggest a social penalty associated with facial paralysis.

## Key Points

**Question:**

How is facial paralysis associated with the perception of attractiveness, femininity or masculinity, and personality, and do patient-reported outcome measures correlate with how patients are perceived by others?

**Findings:**

In this cross-sectional study including 20 patients with facial paralysis and 122 survey respondents, respondents rated photographs of patients with facial paralysis significantly lower in likeability, trustworthiness, attractiveness, and femininity or masculinity compared with the digitally edited images of patients without facial paralysis. Higher social function and total Facial Clinimetric Evaluation scores were associated with increased trustworthiness and attractiveness scores.

**Meaning:**

These results broaden understanding of how facial paralysis is associated with societal perceptions of persona.

## Teaser

This cross-sectional study examines how facial paralysis is associated with perception of attractiveness, femininity or masculinity, and personality.

## Introduction

Facial expressions play an important role in the way we communicate emotions and how people develop impressions of us. When judging everyday images of strangers’ faces, perceivers notably rely on broad and simple facial cues, such as the presence of a smile, to create impressions.^1,2^ The smile is perceived as an indicator of approachability and trustworthiness and can be seen as a signal of warmth and approval in many cultures.^3,4,5,6^ A 2015 study^7^ suggested a relationship between the prevalence of smiling and the amount of immigration in a region. It appears the smile may be part of the universal body language of making a connection, and nowhere is this more apparent than in the US.^7^

One of the key components of a successful smile is dynamic symmetry, such that the left and right sides of the mouth are temporally synced.^8^ However, patients with facial paralysis have a limited degree of excursion on the paralyzed side, impairing affect display.^9,10,11^ Using eye-tracking technology, it has been shown that for observers, the greatest deviation from normal is noted when encountering mouth asymmetries.^11^ Furthermore, there are often unwanted synkinetic movements of the ipsilateral facial musculature after incomplete recovery of facial paralysis, specifically the orbicularis oculus. These aberrant movements likely further contribute to the perceived abnormality. Therefore, it is not surprising that faces of patients with facial paralysis are viewed as significantly more negative, more distressed, and less trustworthy.^12^

In addition to the notable facial asymmetry, the loss of a spontaneous smile and inability to express certain emotions is particularly distressing for patients with facial paralysis. Many patients describe feeling that they are negatively perceived and experience increased social anxiety, depression, and avoidance behaviors.^13,14,15,16,17,18^ These findings are in line with the phenomena of affective realism, whereby an inability to effectively smile can increase risk of anxiety and depression.^19,20,21^ Overall, psychological distress from facial paralysis has been found to be the most significant predictor of social disability.^22^

While it is generally recognized that facial paralysis brings a social penalty, the perceptions of specific personality traits have yet to be quantified, to our knowledge. The purpose of this study is to evaluate and quantify changes in attractiveness, femininity or masculinity, and personality perception of patients with facial paralysis. In addition, we assess the association of perception by others with self-perception with respect to facial impairment. Finally, we explore the variation in the social penalty of facial paralysis as it relates to sex.

## Methods

Ethical approval for this study was obtained from the Georgetown University Medical Center institutional review board. Twenty consecutive patients with a diagnosis of facial paralysis in the MedStar Georgetown University Hospital, Department of Otolaryngology, from January 1, 2014, to December 31, 2016, were enrolled in the study. All patients provided written informed consent, including consent to use digitally altered images of their smiling facial expression. Of note, all photographs were taken during initial evaluation of the patient, using standardized views and lighting. This study is reported following the Strengthening the Reporting of Observational Studies in Epidemiology (STROBE) reporting guideline.

**Figure 1.**
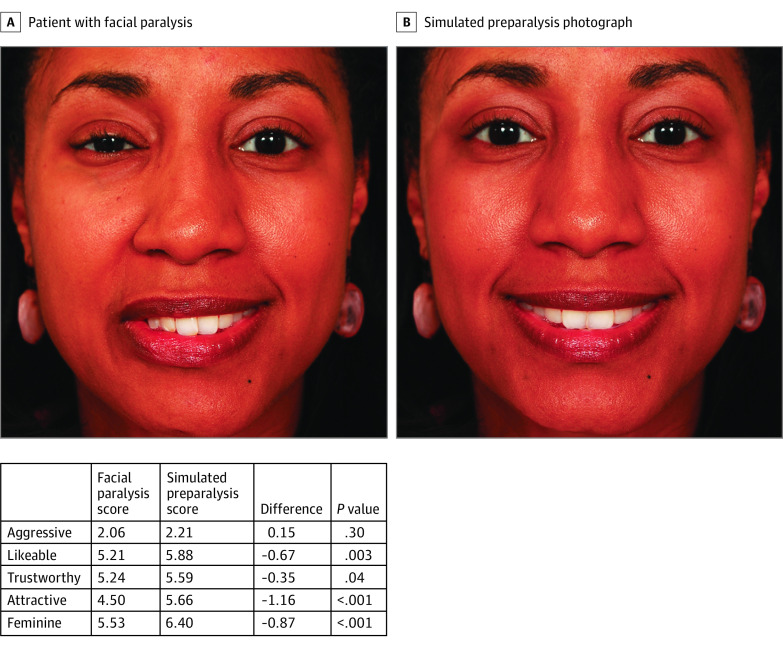
Woman With Facial Paralysis Compared With Her Simulated Preparalysis Photograph and Personality Perception Scores

**Figure 2.**
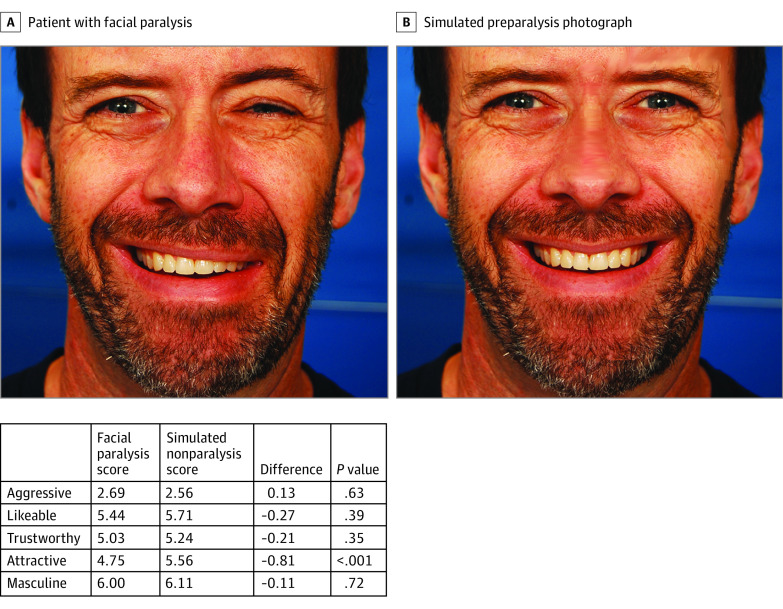
Man With Facial Paralysis Compared With His Simulated Preparalysis Photograph and Personality Perception Scores

The 2 smiling images of each patient (1 unaltered image with facial paralysis and 1 digitally altered image using Adobe Photoshop image editing software with a symmetrical smile) were used. This resulted in 40 total images. Figure 1 and Figure 2 display a women and man patient with facial paralysis and their digitally altered photographs. Four surveys, each consisting of 10 sets of photos (5 graphically altered, 5 unaltered) were constructed. The surveys were designed such that photographs of the same patient were not placed in the same survey to prevent recall bias or direct comparison by survey respondents. Respondents were recruited to participate in this study using the Georgetown University email listserv. Each of these surveys was then administered in person until a minimum of 20 responses were received for each survey. Written informed consent was obtained from all survey respondents.

Survey respondents provided basic demographic information and rated the level to which they believed themselves to be critical of the appearance of others on a 7-point Likert scale. They additionally rated their perception of each pictured patient’s personality traits (ie, aggressiveness, likeability, risk seeking, and trustworthiness), attractiveness, and femininity or masculinity. These personality traits were selected based on previous identification of these traits as having valid and reliable ratability.^23,24,25,26^ Survey respondents were blinded to the study intent. The study excluded respondents with experience in facial analysis or facial plastic surgery.

The Facial Clinimetric Evaluation (FaCE) questionnaire is a validated patient-graded scale that focuses on the functional aspects and quality of life associated with facial paralysis.^27^ It involves 15 statements, each using a 5-item Likert scale, whereby 1 designates the lowest function and 5, the highest. The statements are then grouped into 6 independent domains: social function, facial movement, facial comfort, oral function, eye comfort, and lacrimal comfort. An overall score incorporating all 6 domains is then calculated with results from 0, indicating worst, to 100, best. The FaCE scale has demonstrated high test-retest reliability (Spearman correlation *r* = 0.88; *P* < .01) and internal consistency of the various domains (*r* = 0.81-0.92; *P* < .01).^27,28^ For our analysis, scores from the FaCE questionnaire that patients completed at the time of the initial photographs were also included. Specifically, the social function score as well as the total FaCE score were included to account for self-perception.

### Statistical Analysis

A linear mixed-effect model was applied to analyze patient’s trait data, as this method can efficiently account for the correlation among raters within each patient. Ratings of patient’s traits were summarized as means and SEs. Fisher exact, χ^2^, and Kruskal-Wallis tests were applied to test the difference of respondents’ demographic characteristics between surveys. Spearman correlation statistics were used to assess association of FaCE with various personality traits. Two-sided *P* value and 95% CIs were provided for the group difference and correlation using α = .05 as the cutoff for significance. SAS statistical software version 9.4 (SAS Institute) was used for the analysis. Data were analyzed from November 11, 2019, to February 22, 2020.

## Results

This study included photographs of 20 patients with facial paralysis (mean [range] age, 54 [28-69] years; 15 [75%] women). Most patients with facial paralysis were white (17 patients [85%]) and had facial paralysis as a result of Bell palsy (11 patients [55%]) (Table 1). A total of 122 survey respondents completed 1 of the surveys (71 [61%] women), with most respondents being aged 25 to 34 years (79 respondents [65%]) (Table 2). The total number of respondents for each survey ranged from 27 to 34 respondents. Across all 4 surveys, respondent age, race/ethnicity, and the degree to which survey responders criticized the appearance of others did not vary significantly.

**Table 1.** Demographic Characteristics of Patients With Facial Paralysis

Characteristic	No. (%)
Age, mean (range), y	54 (28-69)
Sex	
Women	15 (75)
Men	5 (25)
Race	
White	17 (85)
African American	3 (15)
Cause of paralysis	
Bell palsy	11 (55)
Acoustic neuroma	3 (15)
Ramsay Hunt syndrome	2 (10)
Parotid carcinoma	2 (10)
Facial nerve schwannoma	2 (10)
Duration of paralysis, mean (range), y	
Overall	4.58 (0.73-9.08)
Women	4.95 (0.73-9.08)
Men	3.47 (1.32-4.87)
Social perception score, mean (range)	
Overall	71.10 (6.25-87.50)
Women	73.43 (6.25-87.50)
Men	64.06 (10.00-80.00)
Total FaCE score, mean (range)	
Overall	57.19 (30-85)
Women	58.72 (32-85)
Men	50.56 (30-72)

Abbreviation: FaCE, Facial Clinimetric Evaluation.

**Table 2.** Demographic Characteristics of Survey Respondents

Characteristic	No. (%)
Age, y	
18-24	13 (11)
25-34	79 (65)
35-44	10 (8)
45-54	9 (7)
55-64	8 (7)
65-74	2 (2)
≥75	1 (1)
Sex	
Men	47 (38.52)
Women	75 (61.48)
Race/ethnicity	
White	87 (72)
Asian	21 (17)
African American	6 (5)
Other	6 (5)
Native Hawaiian or Pacific Islander	1 (1)
Education	
Bachelor’s degree	68 (56)
Graduate degree	51 (42)
Some college	3 (2)

Patients with facial paralysis were perceived as less likeable (mean difference, −0.29; 95% CI, −0.43 to −0.14), trustworthy (mean difference, −0.25; 95% CI, −0.39 to −0.11), attractive (mean difference, −0.47; 95% CI, −0.62 to −0.32), and feminine or masculine (mean difference, −0.21; 95% CI, −0.38 to −0.03) compared with their digitally altered photographs (Table 3). When divided by sex, we found that women with facial paralysis were perceived as significantly less likeable (mean difference, −0.34; 95% CI, −0.53 to −0.16), trustworthy (mean difference, −0.24; 95% CI −0.43 to −0.06), attractive (mean difference, −0.74; 95% CI, −0.94 to −0.55), and feminine (mean difference, −0.35; 95% CI, −0.58 to −0.13) compared with their digitally altered photographs. Men were only perceived to be significantly less likeable (mean difference, −0.24; 95% CI, −0.47 to −0.01) and trustworthy (mean difference, −0.30; 95% CI, −0.53 to −0.07) compared with their digitally altered photographs.

**Table 3.** Comparison of Rating Between Simulated Preparalysis and Facial Paralysis Groups Using Linear Mixed-Effect Model

Trait	Estimate (SE)			95% CI	*P* value
	Simulated preparalysis	Facial paralysis	Difference		
Aggressive	2.99 (0.15)	3.02 (0.15)	0.03 (0.09)	−0.14 to 0.20	.74
Likeable	4.98 (0.13)	4.69 (0.13)	−0.29 (0.07)	−0.43 to −0.14	<.001
Trustworthy	4.75 (0.11)	4.50 (0.11)	−0.25 (0.07)	−0.39 to −0.11	<.001
Attractive	4.13 (0.18)	3.66 (0.18)	−0.47 (0.08)	−0.62 to −0.32	<.001
Feminine or masculine	5.18 (0.18)	4.98 (0.18)	−0.21 (0.09)	−0.38 to −0.03	.02
**Women**					
Aggressive	2.92 (0.14)	2.91 (0.14)	−0.009 (0.12)	−0.24 to 0.22	.94
Likeable	5.15 (0.13)	4.81 (0.13)	−0.34 (0.10)	−0.53 to −0.16	<.001
Trustworthy	4.81 (0.11)	4.56 (0.11)	−0.24 (0.09)	−0.43 to −0.06	.01
Attractive	4.36 (0.19)	3.61 (0.19)	−0.74 (0.10)	−0.94 to −0.55	<.001
Feminine	5.25 (0.19)	4.90 (0.19)	−0.35 (0.11)	−0.58 to −0.13	.002
**Men**					
Aggressive	3.01 (0.18)	3.12 (0.18)	0.11 (0.14)	−0.17 to 0.39	.45
Likeable	4.74 (0.15)	4.51 (0.15)	−0.24 (0.12)	−0.47 to −0.01	.04
Trustworthy	4.70 (0.12)	4.40 (0.12)	−0.30 (0.12)	−0.53 to −0.07	.01
Attractive	3.78 (0.20)	3.69 (0.20)	−0.090 (0.12)	−0.32 to 0.14	.45
Masculine	5.14 (0.20)	5.04 (0.19)	−0.095 (0.14)	−0.38 to 0.18	.50

When correlating patients’ self-perception of their social function with various personality traits, we found that as social function scores increased, there was a significant increase in the perception of trustworthiness (*r*_s_[480] = 0.12; *P* = .01). In analysis including the total FaCE score, there was a significant association between increased scores and perceived trustworthiness (*r*_s_[480] = 0.11; *P* = .02) and attractiveness (*r*_s_[478] = 0.10; *P* = .04) (eTable in the Supplement).

## Discussion

The Duchenne smile is classically described as the anatomical marker of the genuine smile. The smile is distinctive, with the mouth turning up from the activation of the zygomatic major muscle, the cheeks lifting, and the appearance of wrinkles around the eyes (also known as *crow’s feet*) associated with simultaneous contraction of the orbicularis oculi. The absence of the Duchenne smile not only influences how people evaluate smiles but also how they are judged by others.^29,30^

The findings of this cross-sectional study suggest that the inability to effectively smile is associated with negative perceptions in likeability, trustworthiness, attractiveness, and femininity or masculinity for patients with facial paralysis. Paralysis affecting the mouth is among the most notable of facial asymmetries, such that palsies of the zygomatic and marginal branches of the facial nerve are considered to have a significantly greater need for correction.^11,12^ Interestingly, reanimation surgery of the lip significantly decreases the degree of attention to the mouth and can help decrease negative perceptions of patients with facial paralysis.^31^

A universal finding for our patient population was lower perceived trustworthiness for the photographs of patients with facial paralysis vs their digitally altered counterparts. Research in the psychological and social sciences corroborate these findings, such that a happy facial expression makes a person appear more trustworthy.^31,32^ Furthermore, having a facial appearance that conveys a positive emotional state enhances trust.^33,34,35^ These findings highlight the social significance of the asymmetric smile and the importance of further progress in the development of techniques to assist in mitigating the effects of facial paralysis.

It is interesting to find that men and women with facial paralysis did not experience the same social penalty with respect to their facial paralysis. The relative decrease in attractiveness and femininity perceived in women with facial paralysis likely reflects the different social expectations by sex in our society. This is consistent with the results reported in a 2019 study^36^ that suggest that the appearance of a smile is not as integral to the perception of masculinity as it is to femininity.

Lastly, there was a correlation between the way patients with facial paralysis perceived themselves and how they were perceived by others. Specifically, as self-perception of social function and overall facial function improved, there was an increase in perceived trustworthiness and attractiveness by others. This is similar to the results reported by Lyford-Pike et al^37^ that suggest that higher FaCE scores correspond with decreased perception of disfigurement by patients.

It is important to note that this study included patients with facial paralysis presenting with a range of facial impairment. Not all patients with facial paralysis experienced a significant decrease in the perception of their personality traits, femininity or masculinity, and attractiveness. More research is needed to better understand the different variables that can optimize outcomes at the individual patient level.

### Limitations

There are several limitations to this study. This study was performed using static smiling images, but other studies have found that observers judged the severity of paralyzed faces to be more noticeable when viewing dynamic expressions.^38^ In addition, as this study included only patients willing to have their photos viewed by others, there may have been a selection bias rendering the study patient group to be less reflective of the true gamut of patients with facial paralysis.^39^

## Conclusions

The findings suggest that facial paralysis was associated with worse outcomes in the way patients were perceived in terms of likeability, trustworthiness, attractiveness, and femininity or masculinity. Furthermore, improved self-perception based on the FaCE questionnaire was correlated with an increased perception of trustworthiness and attractiveness by others. Further research is warranted to determine the degree to which specific sequelae of facial paralysis may affect the perception of various personal qualities and how therapeutic interventions may mitigate these effects.

## Notes

Supplement. **eTable.** Spearman Correlation Statistics for Social Function and Total Facial Clinimetric Evaluation Scores With Perceptions of Personality Traits
