# Supplementary material for: Association of Facial Paralysis With Perceptions of Personality and Physical Traits
Source: JAMA Netw Open. 2020 Jun 24;3(6):e205495. doi: 10.1001/jamanetworkopen.2020.5495 (PMC7315303; doi:10.1001/jamanetworkopen.2020.5495)
Supplement: Supplement. — eTable. Spearman Correlation Statistics for Social Function and Total Facial Clinimetric Evaluation Scores With Perceptions of Personality Traits [file jamanetwopen-3-e205495-s001.pdf]

# Supplementary Online Content

Parsa KM, Hancock M, Nguy PL, et al. Association of Facial Paralysis With Perceptions of Personality and Physical Traits. *JAMA Netw Open*. 2020;3(6):e205495. doi:10.1001/jamanetworkopen.2020.5495

**eTable.** Spearman Correlation Statistics for Social Function and Total Facial Clinimetric Evaluation Scores With Perceptions of Personality Traits

This supplementary material has been provided by the authors to give readers additional information about their work.

© 2020 Parsa KM et al. *JAMA Network Open.*

eTable. Spearman Correlation Statistics for Social Function and Total Facial Clinimetric Evaluation Scores With Perceptions of Personality Traits

| Variable | With Variable | N | Correlation | Fisher's z | 95% Confidence Limits | | P-value |
|---|---|---|---|---|---|---|---|
| **Social Function** | Aggressive | 480 | -0.037 | -0.037 | -0.126 | 0.052 | 0.41 |
| | Likeable | 479 | 0.018 | 0.018 | -0.071 | 0.108 | 0.68 |
| | Trustworthy | 480 | 0.120 | 0.121 | 0.031 | 0.207 | 0.01 |
| | Attractive | 478 | 0.075 | 0.075 | -0.014 | 0.164 | 0.09 |
| | Femininity/Masculinity | 366 | -0.015 | -0.015 | -0.117 | 0.087 | 0.76 |
| **Total FaCE** | Aggressive | 480 | -0.032 | -0.032 | -0.121 | 0.057 | 0.48 |
| | Likeable | 479 | 0.031 | 0.030 | -0.059 | 0.119 | 0.50 |
| | Trustworthy | 480 | 0.106 | 0.106 | 0.016 | 0.193 | 0.02 |
| | Attractive | 478 | 0.091 | 0.092 | 0.002 | 0.180 | 0.04 |
| | Femininity/Masculinity | 366 | 0.014 | 0.014 | -0.088 | 0.116 | 0.78 |

© 2020 Parsa KM et al. *JAMA Network Open.*
